# Diuretics, SGLT2 inhibitors and falls in older heart failure patients: to prescribe or to deprescribe? A clinical review

**DOI:** 10.1007/s41999-023-00752-7

**Published:** 2023-02-03

**Authors:** Eveline P. van Poelgeest, M. Louis Handoko, Majon Muller, Nathalie van der Velde, On behalf of the EUGMS Task & Finish group on Fall-risk-increasing drugs

**Affiliations:** 1 Department of Internal Medicine/Geriatrics, Amsterdam University Medical Centers, Location University of Amsterdam, Meibergdreef 9, Amsterdam, The Netherlands; 2 Amsterdam Public Health Research Institute, Aging and Later Life, Amsterdam, The Netherlands; 3 Department of Cardiology, Amsterdam University Medical Centers, De Boelelaan 1117, Amsterdam, The Netherlands; 4 Amsterdam Cardiovascular Sciences Institute, Amsterdam, The Netherlands

**Keywords:** Deprescribing, Diuretics, Falls, Geriatric, Sodium-glucose cotransporter-2 inhibitors

## Abstract

**Purpose:**

Both heart failure and its treatment with diuretics or SGLT2 inhibitors increase fall risk in older adults. Therefore, decisions to continue or deprescribe diuretics or SGLT2 inhibitors in older heart failure patients who have fallen are generally highly complex and challenging for clinicians. However, a comprehensive overview of information required for rationale and safe decision-making is lacking. The aim of this clinical review was to assist clinicians in safe (de)prescribing of these drug classes in older heart failure patients.

**Methods:**

We comprehensively searched and summarized published literature and international guidelines on the efficacy, fall-related safety issues, and deprescribing of the commonly prescribed diuretics and SGLT2 inhibitors in older adults.

**Results:**

Both diuretics and SGLT2 inhibitors potentially cause various fall-related adverse effects. Their fall-related side effect profiles partly overlap (e.g., tendency to cause hypotension), but there are also important differences; based on the currently available evidence of this relatively new drug class, SGLT2 inhibitors seem to have a favorable fall-related adverse effect profile compared to diuretics (e.g., low/absent tendency to cause hyperglycemia or electrolyte abnormalities, low risk of worsening chronic kidney disease). In addition, SGLT2 inhibitors have potential beneficial effects (e.g., disease-modifying effects in heart failure, renoprotective effects), whereas diuretic effects are merely symptomatic.

**Conclusion:**

(De)prescribing diuretics and SGLT2 inhibitors in older heart failure patients who have fallen is often highly challenging, but this clinical review paper assists clinicians in individualized and patient-centered rational clinical decision-making: we provide a summary of available literature on efficacy and (subclass-specific) safety profiles of diuretics and SGLT2 inhibitors, and practical guidance on safe (de)prescribing of these drugs (e.g. a clinical decision tree for deprescribing diuretics in older adults who have fallen).

## Key summary points

**Aim:**

This study aimed at summarizing the available literature on fall risk associated with diuretic and SGLT2i use in older heart failure patients, including the underlying pathophysiology, and to assist clinicians in safe (de)prescribing of these drug classes.

**Findings:**

Both heart failure and diuretic or SGLT2 inhibitor use increase fall risk in older adults. Diuretics and SGLT2 inhibitors not only have various fall-related adverse effects, which partly overlap (e.g. tendency to cause hypotension), but also differ: compared to SGLT2is, diuretics display more fall risk factors (e.g. electrolyte abnormalities). The tendency to cause fall-related adverse effects may differ according to diuretic sub-class or even within sub-classes.

**Message:**

Decisions to continue or deprescribe diuretics or SGLT2is in fall-prone older HF patients are generally highly complex, but detailed insight into fall-related side effect profiles of these drug classes, and practical clinical decision tools and resources, assist prescribers in rational and personalized (de)prescribing.

## Case illustration

A 83-year-old woman visits the falls clinic for analysis of recurrent falls. Multifactorial falls risk assessment (including comprehensive geriatric assessment [1]) reveals a clinical frailty score of 5, HFpEF with an episode of congestive heart failure three years ago, hypertension, type 2 diabetes with end-organ damage, CKD G3b, and osteoporosis with a recent hip fracture. She has poor sleep due to nocturia, and is afraid of falling, causing her to limit physical activities and to avoid social events. Her most important goal of care is to not fall anymore, and to keep living independently. Her medication use includes enalapril, furosemide twice daily 40 mg, hydrochlorothiazide once daily 12.5 mg, long-acting metoprolol once daily, 10 units of long-acting insulin, metformin, calcium, vitamin D, denosumab and temazepam. She uses two units of alcohol daily. Blood pressure is RR 128/89 mmHg, heart rate 89 beats per minute, and BMI 35 kg/m^2^. She has orthostatic complaints and initial orthostatic hypotension. Laboratory testing: serum creatinine 118 µmol/L, BUN 15 mEg/L, eGFR 32 mL/min/1.73 m^2^, sodium 132 mEq/L, potassium 4.2 mEq/L, HbA1c 70 mmol/mol (8.6%)).

## Introduction

Every year in the European Union, at least 3.8 million older adults visit emergency departments for fall-related injuries, of which 1.4 million require hospital admission [2]. Besides the need for hospital treatment, falls negatively affect functionality and quality of life in this age group [3]. Older patients with a history of heart failure (HF) represent a notoriously vulnerable patient population, for which optimal care by nature is holistic (multidomain) [4–6]. Because of combinations of fall-risk-increasing conditions including frailty, cognitive impairment, depression, cachexia and/or sarcopenia, anxiety and insomnia, older HF patients are at very high fall risk (43% fall in a 2-year period) [7]. Diuretics are considered important fall-risk-increasing drugs (FRIDs). In older adults with age-related attenuated baroreceptor function, and impaired ability to adequately maintain intravascular volume, the diuretic effect may cause or aggravate fall risk [8]. To mitigate fall risk, the World guidelines for falls prevention and management for older adults [1] recommend considering deprescribing FRIDs.

In patients with a history of HF, however, deprescribing diuretics may not be an effective fall preventive intervention as it may cause congestive HF, in itself an independent fall risk factor [9]. On the other hand, some small studies show that in selected patients, diuretic withdrawal in older adults is safe and feasible [10, 11]. In-depth knowledge on patient selection, and the risks and benefits of prescribing and deprescribing of diuretics in fall-prone older HF patients is currently lacking. Yet, this knowledge is necessary for clinicians who care for older fall-prone HF patients.

In this clinical review, we aim to provide the reader with a summary of the evidence base for diuretics in older HF patients, a summary of the literature on deprescribing these drug classes, and an overview of their potential fall-related side effects. In addition, we will also discuss the relatively new SGLT2i drug class: like diuretics, SGLT2is have diuretic effects and are recommended in HF treatment. Over the past years, the use of SGLT2is has increased rapidly in all age groups, including the oldest adults [12]. Given the prominent positions in (inter)national guidelines, it is likely that the use of SGLT2is in this population will continue to increase. This paper will assist clinicians in making informed, individualized decisions about when and how to safely continue, taper, stop, or switch diuretics and SGLT2is to safer alternatives in older persons at increased risk of falling.

## Methods

This narrative review summarizes the currently available evidence on both benefits and fall-related harms of chronic use and deprescribing diuretic and SGLT2i formulations in older HF patients. The review was informed by a literature search conducted in November 2022 in Medline and Google Scholar with keywords “falls”, “diuretic”, “SGLT2 inhibitor”, “deprescribing” and “older adults”. Personal reference libraries and diuretic/SGLT2i SmPCs (summary of product characteristics) were also utilized. We included the most commonly prescribed diuretics and SGLT2is. Advanced, non-pharmacological HF therapies (e.g. cardiac resynchronization devices) were beyond the scope of this review. We screened titles and abstracts of potentially relevant articles in English language and included based on relevance to the aim of the project. We performed citation and reference checking of the included papers. Based on availability, we selected the most recently published and/or highest quality evidence. Consistent with the narrative review methodology, we did not appraise methodological quality or risk of bias of the included articles.

## Matching diuretic or sglt2i use to appropriate indication

### Appropriate prescribing of diuretics and SGLT2is

Diuretics are the cornerstone of congestion treatment in HF (2021 ESC HF guideline [13], grade 1C). They reverse congestion with volume overload by diminishing sodium reabsorption at different sites in the nephron, leading to increased renal natriuresis and diuresis [14], thereby reducing intracardiac pressures and improving cardiac performance [15]. Diuretics alleviate HF symptoms (such as dyspnea and exercise intolerance), improve exercise capacity and reduce HF hospitalizations/mortality irrespective of type of HF [13, 16–18]. If inadequately treated, (residual) congestion may lead to loss of appetite/weight loss [17] and early (re)hospitalization. The latter is especially important to avoid in older adults as hospital admissions in this patient group are associated with functional decline and iatrogenic harm.

SGLT2is are recommended as first line disease-modifying treatment in chronic HFrEF to reduce the risk of HF hospitalization and death according to the 2021 ESC HF guideline (IA evidence for empagliflozin and dapagliflozin) [13] alongside RAASis/ARNIs, beta blockers and MRAs [19, 20]. By causing (mild) diuresis, SGLT2is decrease systemic, venous and pulmonary overload as well as extracellular edema. This results in improved end-diastolic pressure, decreased ventricular wall stress and increased cardiac output. These drugs have proven benefits regarding MACE (major adverse cardiovascular event) outcomes (e.g. myocardial infarction and cardiovascular mortality), especially in older patients [21]. Data from large clinical trials published after the 2021 ESC guideline demonstrate that SGLT2is are also safe and efficacious in HFpEF. In fact, empagliflozin has recently been approved by the FDA and EMA for the treatment of chronic HF across all categories of HF. In high-risk patients (diabetes mellitus type 2 and/or cardiovascular disease), SGLT2is are recommended to prevent HF hospitalizations. Hospitalization risk reduction is already observed within 2 weeks after SGLT2i initiation [22]. Initiation of empagliflozin in (relatively young) older adults (68.5 ± 13.3 years) resulted in a 36% clinical benefit (composite of all-cause death, HF events and symptom score) compared to placebo, apparent already within 15 days after SGLT2 initiation [23].

### (Potentially) inappropriate prescribing of diuretics and SGLT2is

Whereas diuretics are effective cornerstones for symptom control in patients with systolic HF and congestion, they lack disease-modifying effects. Chronic, long-term diuretic therapy to prevent congestion is not supported by high-quality evidence, and safety in outpatient settings is controversial [17]. Inappropriate diuretic prescription in non-congested patients leads to preload reduction and lower cardiac output, provoking or aggravating exercise intolerance, fatigue [10] and orthostatic hypotension (OH) [10]. In addition, it may result in sympathetic overactivity, which is associated with worse prognosis in older community-dwelling adults, and patients with HF or end stage renal disease [22]. Real-world data show that inappropriate diuretic use is highly prevalent in older patients. For example, a recent study in a nursing home population showed that approximately one fifth of older diuretic users did not have a cardiovascular diagnosis [24]. In a recent Dutch trial on unplanned hospital admissions (*n* = 16,687) in older patients, the prevalence of inappropriate loop diuretic prescriptions (for example for venous insufficiency) was 11% [25]. Frequently, diuretics are continued for years without withdrawal attempts after a first episode of HF that was caused by an underlying condition that may have been resolved (e.g., acute ischemic or arrhythmic event). Alternatively, the diuretic may be part of a prescribing cascade. In fact, one of the most common prescribing cascades is initiation of a loop diuretic for ankle edema after starting a calcium channel blocker (e.g., amlodipine) that is erroneously considered to be caused by new-onset HF instead of a drug side effect [26]. Often overlooked, but also relevant to consider in this regard is the gabapentinoid–oedema–loop diuretic prescribing cascade [27]. Not only is diuretic therapy not indicated in this setting, but it may also even be harmful by causing neurohumoral activation leading to secondary aldosteronism and sodium retention.

SGLT2is augment the diuresis effect caused by diuretics [28]. This may cause or aggravate (orthostatic) hypotension and acute kidney injury in frail older adults, and in non-frail older adults in case of conditions leading to dehydration (e.g. vomiting and diarrhea). In addition, given their side effect profile [29], SGLT2i therapy may be inappropriate in patients at risk for genitourinary tract infections, euglycemic DKA and possibly (demonstrated solely for canagliflozin) lower-extremity amputations and fractures. SGLT2is should be prescribed in these individuals at risk with great caution. Doctors should closely monitor for these complications, and if they occur reconsider continuation therapy [30].

## Diuretic and SGLT2i pharmacology

Diuretics are heterogeneous in their pharmacological properties [31]. As an example, loop diuretics have steep dose–response curves (below a given threshold plasma concentration there is little natriuretic or diuretic effect). After uptitrating loop diuretic dose to a certain plasma concentration, a plateau (“ceiling”) is reached, and further increase in plasma concentrations will fail to increase natriuresis and diuresis [31]. In contrast, thiazide diuretics have a shallow dose–response curve (there is little difference between the lowest and maximal effective dose) [32]. Pharmacological properties may also differ within diuretic subclasses: oral bioavailability for furosemide ranges from 10 to 100% and is > 80% for torasemide and bumetanide [33]. The duration of effect of torasemide (6–16 h) is relatively long compared to that of bumetanide (4–6 h) and furosemide (6–8 h). Also, inter-individual differences based on genetic polymorphisms and sex differences in diuretic efficacy and tendency to develop adverse effects have been demonstrated [34–36].

Pharmacokinetic and pharmacodynamic profiles of the SGLT2is as a group are comparable, and favorable for older individuals as follows: oral bioavailability is generally good, elimination half-life relatively long (allowing once-daily dosing), renal excretion limited and tendency to cause drug–drug interactions low [37]. Of note, however, SGLT2is tend to decrease body weight with 1.4–4 kg [38]. After an initial weight loss resulting from diuretic fluid loss, most of the subsequent weight loss associated with SGLT2i appears to be driven by a loss in fat mass [39].

### Dosing of diuretics and SGLT2is

Diuretic use is associated with dose-dependent, severe, potentially life-threatening adverse effects. In fact, up-titrating diuretics is associated with worsening renal function, increased hospital admission rates and mortality [17]. Fall risk associated with diuretic use is significantly elevated the first 3 weeks after diuretic initiation, switch or dose increase [40]. Kidney function decline is one of the most predictive markers for adverse outcomes in HF [41]. Therefore, the goal of diuretic therapy in HF management is to prescribe the lowest effective dose to reach and maintain euvolemia [41, 42] for the individual patient [17, 43]. However, identification of a patients’ optimal dose may be challenging, especially in case of cognitive decline and limited physical activity [17]. International guideline-based congestion management recommendations for chronic HF patients are scarce and not very detailed. However, a practical expert-based congestion treatment algorithm has recently been published [17].

After successfully treating congestion, guidelines recommend down-titration of loop diuretics in chronic HF when patients have no signs of volume overload [44]. It is estimated that diuretic doses for (acute) recompensation are two- to threefold the dose required as maintenance therapy [45]. Thus, an attempt to reduce diuretic dose by 50% seems rational [17]. Furosemide dose lowering in older patients with stable systolic HF and underlying renal dysfunction was demonstrated to be safe and associated with an improvement in GFR without a change in volume or functional status [41].

In contrast to diuretics, SGLT2is do not require identification of individualized optimal dose (they are fixed-dose) and appear to be devoid of a tendency to cause acute kidney injury (AKI) [46] or kidney function decline (see next paragraph).

### Diuretics and SGLT2is and kidney function decline

Chronic kidney disease is a common comorbidity in HF patients, especially in HFrEF [47]. When present, it is associated with worse HF severity and poor cardiovascular outcomes. Patients with CKD are often diuretic resistant, for example due to poor drug absorption, and impaired tubular secretion and compensatory sodium reabsorption from unblocked sodium transporters [48]. Loop diuretics are recommended for patients with eGFR ≤ 30 mL/min/1.73 m^2^. When GFR falls below 30 mL/min, thiazide diuretics are unable to achieve sufficient concentrations to exert their action in the distal tubule [49]. Although addition of thiazide diuretics to loop diuretics has been shown to promote natriuresis and decongestion in loop diuretic–resistant patients, long-term benefit in reducing cardiovascular mortality remains uncertain, and electrolyte disorders are common.

In contrast to diuretics, SGLT2is maintain natriuresis in patients with reduced eGFR [48]. Although current international guidelines do not recommend the use of SGLT2is in patients with eGFR values ≤ 30 mL/min per 1.73 m^2^, evidence is rapidly emerging that SGLT2is are safe and efficacious in chronic kidney disease patients with lower eGFR (empagliflozin has been studied in patients with eGFR ≥ 20 mL/min per 1.73 m^2^) [47, 50, 51]. Because SGLT2is have kidney protective effects (they slow kidney disease progression), it is likely that in the near future, there will be a shift toward more liberal use in chronic kidney disease [52]. It should be noted that SGLT2is (like other drugs in HF treatment, such as ACEi/ARB and ARNi) result in an initial decline in eGFR that generally stabilizes over time. As such, this pseudo-kidney function worsening can be accepted without dose-reduction or discontinuation, unless the patients’ clinical condition worsens (Table 1) [47].

**Table 1:** Practical tools and resources for patient-centered safe (de)prescribing of diuretics and/or SGLT2is in older heart failure patients

Tool or resource	Clinical application
ADA (American Diabetes Association) recommendations on SGLT2i treatment in frail individuals [21, 59]	Frailty-status stratified treatment recommendations
Barcelona Bio-HF Calculator Version 2.0	An individualized risk estimate calculator for 1-, 2-, 3-, 4- and 5-year mortality, heart failure -related hospitalization, and the composite endpoint
Expert-based congestion treatment algorithm [17]	Practical algorithm for managing congestion
LIFE-HF calculator, a lifetime risk calculator for heart failure patients (*Submitted and presented at the 2022 ESC congress*)	A patient-friendly tool for showing an estimation of the number of years gained free of cardiovascular death or hospitalizations after initiating various HF medications
Recommendations for dealing with (pseudo)worsening of kidney function in HF patients after initiating SGLT2i therapy [47]	Practical guidance for (pseudo)worsening of kidney function upon initiating drugs (incl SGLT2is) in HFrEF patients with CKD
STOPPFall Supplementary Table 7. Diuretics (https://kik.amc.nl/falls/decision-tree/)	Practical decision tree for diuretic withdrawal in patients who have fallen, including guidance on rate of dose-reduction, and how to monitor

## Fall-related adverse effects of diuretics and SGLT2is

**Fig. 1:**
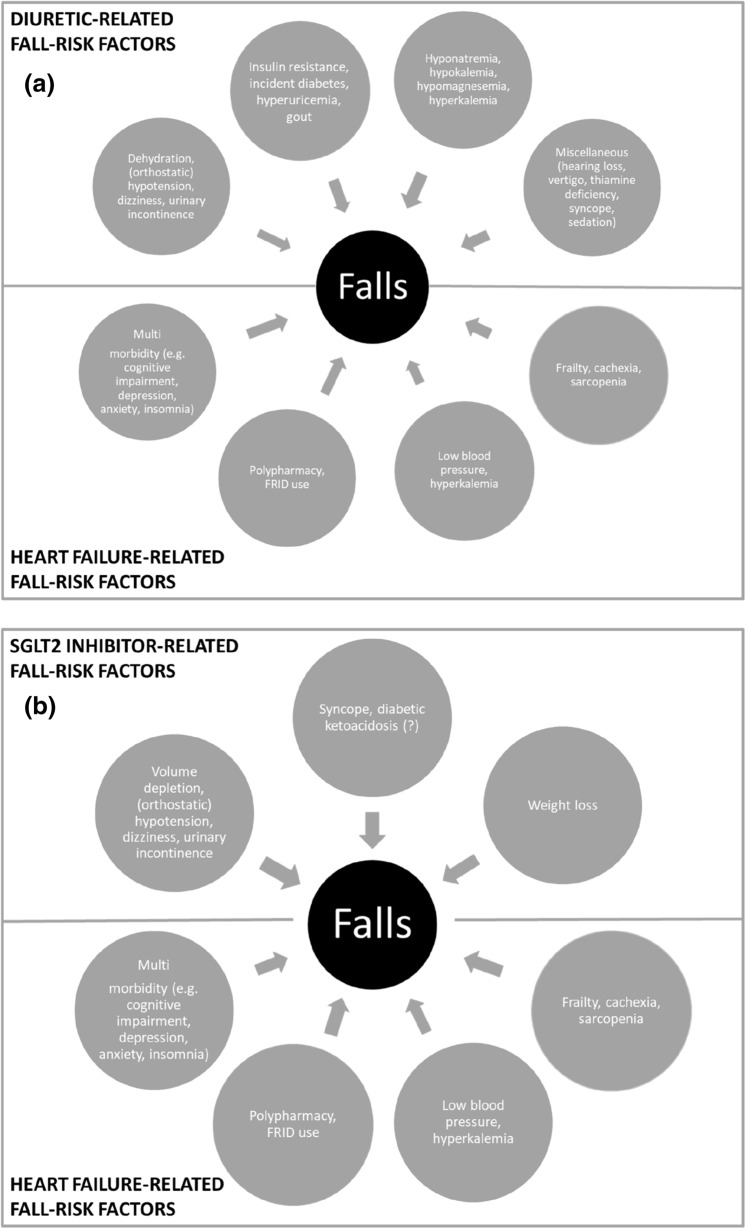
**a** Diuretic-related and heart failure related fall risk factors. **b** SGLT2i-related and heart failure related fall risk factors

Both diuretics and SGLT2is exhibit effects with a direct or indirect link to falls (e.g. related to electrolyte disorders [53] or OH), but profiles between drug classes differ, not only between diuretics versus SGLT2is, but also between and even within diuretic subclasses (Table 2). As can be seen in Fig. 1, and based on the current evidence base, diuretics as a group appear to have a higher number of potential fall-related adverse effects than SGLT2is. For example, diuretic use may result in (potentially dangerous) electrolyte disorders, whereas this risk with SGLT2i use appears low [38]. In general, loop diuretics are more fall risk increasing than other diuretic subclasses [54]. The combination of specific patient characteristics and the (sub)class differences in fall-related side effect profiles may guide selection of the most appropriate drug for individual patients.

**Table 2:** Prevalence of fall-related side effects of diuretics and SGLT2 inhibitors

	(Orthostatic) Hypotension	Dizziness	Hypokalemia	Hyponatremia	Volume depletion	Sedation	Syncope
Loop diuretics							
Bumetanide (C03CA02)	++	+++	+++	+++	++	+++	++
Furosemide (C03CA01)	++	Not known	Not known	Not known	++	Not known	Not known
Torasemide (C03CA04)	Not known	+++	Not known	+++	+++	Not known	Not known
Thiazide(like) diuretics							
Hydrochlorothiazide (C03AA03)	+++	+	++++	++++	Not known	Not known	Not known
Indapamide (C03BA11)	+	+	Not known	Not known	Not known	Not known	Not known
Aldosterone receptor antagonists							
Eplerenone (C03DA04)	+++	++	Not known	++	++	Not known	++
Spironolactone (C03DA01)	Not known	++	Not known	Not known	Not known	++	Not known
SGLT2 inhibitors							
Canagliflozine (A10BK02)	++	++	Not known	Not known	++	Not known	++
Dapagliflozine (A10BK01)	+++	+++	Not known	Not known	++	Not known	Not known
Empagliflozine (A10BK03)	++	++	Not known	Not known	++	Not known	++
Ertugliflozine (A10BK04)^#^	+ ++	+++	Not known	Not known	+++	Not known	+++

+ : Seldom (< 1/1000)

+ + : Sometimes (1/100–1/1000)

+ ++ : Often (1/10–1/100)

+ + + + : Very often (> 1/10)

^#^Both SGLT2 and SGLT1 inhibitor

Source: diuretic and SGLT2 SmPCs

In this paragraph, we provide a narrative summary of the available evidence regarding fall-related adverse effects of diuretics and SGLT2is.

### (Orthostatic) hypotension and diuresis

Low blood pressure is linked to falls [9] through acute transient cerebral hypoperfusion (e.g., in OH or stroke), neurodegenerative lesions to areas of the brain that govern balance/gait, or cognitive impairment. Blood pressure reduction resulting from thiazide use is more pronounced than that with loop diuretic use in patients with normal kidney function [49]. In advanced HF, baseline blood pressure is often low, even in patients with a history of hypertension [55]. OH is characterized by an impaired blood pressure response to standing and is linked to increased risk of adverse outcomes such as falls, syncope, cardiovascular events (e.g. hospitalization for HF, stroke), cognitive impairment, and mortality [56, 57]. Increased diuresis predisposes to urinary incontinence [21], another independent fall risk factor [58], which potentially negatively impacts quality of life of older individuals [45, 59]. In general, intensive blood pressure control compared with standard treatment in non-frail older individuals does not exacerbate OH, nor increases injurious fall risk [60]. However, this may differ inter-individually. And importantly, in frail older adults at risk of falling, intensive blood pressure treatment may substantially increase (injurious) fall risk [61, 62].

Orthostatic syncope is common, especially in older adults. In older patients living with dementia, orthostatic syncope is the leading cause of syncope [63]. A systematic medication review and reappraisal of drugs potentially responsible for OH in this patient group is, therefore, crucial [64]. Diuretics are among the drugs most frequently responsible for drug-related OH [65]. They induce volume depletion by sodium excretion, favoring OH [56], especially in older adults due to age-related slowing of the baroreceptor response and decreased thirst sensation [38, 66]. Loop diuretics produce a more intense and shorter diuresis than thiazides [13]. They decrease intravascular volume and increase venous pooling, reducing venous return and cardiac output [65]. Thiazides have a moderate blood pressure lowering effect [49]. Among the thiazide-like diuretics, chlorthalidone appears relatively potent [49]. In a recent head-to-head study, however, chlorthalidone was not superior to hydrochlorothiazide for most outcomes [67]. Mineralocorticoid antagonists have a relatively potent antihypertensive effect [49].

SGLT2is cause both natriuresis and osmotic diuresis [68], but the diuretic action of SGLT2is is rather weak [39]. Blood pressure reduction is generally only modest (2–5 mmHg drop in systolic pressure) [38], without causing sympathetic overactivity. In individuals with very high blood glucose levels, however, the drops in blood pressure may be more pronounced and contribute to hypotension and falls [21]. Literature linking SGLT2is to OH is scarce, but a recent preclinical trial demonstrated that SGLT2i use may improve baroreflex sensitivity [69], suggesting a protective instead of worsening effect on OH. In euvolemic patients on a stable diuretic regimen, addition of SGLT2i may necessitate (or facilitate) down-titration or discontinuation of concomitant antihypertensives and diuretics [38, 48].

### Metabolic derangements

#### Hyper- and hypoglycemia

Most loop and thiazide diuretics are associated with insulin resistance and a dose-related increase in incident diabetes risk [70]. Hyperglycemia theoretically contributes to fall risk because of osmotic diuresis leading to dehydration and neurocognitive symptoms. Insulin resistance has been demonstrated for both loop and thiazide diuretics [48]. In contrast to diuretics, SGLT2is mitigate diuretic related hyperglycemia [48]. Although SGLT2i use has been linked to the occurrence of diabetic ketoacidosis in case reports, the incidence of DKA in large clinical trials among type 2 diabetes patients has been consistently low, and not related to age [38]. To minimize the risk of developing DKA in patients using SGLT2is, excessive alcohol ingestion and ketogenic diets should be avoided, and not introduced in patients with type 1 diabetes or a history of DKA [38].

Even though SGLT2is cause glycosuria, the risk of developing hypoglycemia with these drugs appears to be low and not age-dependent [29], even in non-diabetics [38]. Based on trial data, the incidence of diabetic ketoacidosis with SGLT2i use in type 2 diabetics is low and did not appear to increase according to age [29].

#### Hyperuricemia and gout

Hyperuricemia and gout attacks are among the most prevalent adverse effects of diuretic therapy [59], especially loop and thiazide (like) diuretics [71]. Gout attacks are associated with debilitating pain, reduced exercise capacity, and worsening of diastolic dysfunction and long-term prognosis [43]. Theoretically, diuretic-associated gout attacks may contribute to falls, because lower extremity musculoskeletal pain is linked to increased fall risk [72].

Whereas diuretics increase the risk of developing hyperuricemia and gout flares [73], SGLT2is reduce uric acid blood concentrations [48] by stimulating uric acid excretion by the GLUT9 isoform 2 on the apical membrane of the proximal tubule, and possibly by blocking uric acid reabsorption in the collecting duct [74]. A recent systematic review and meta-analysis in type 2 diabetic patients [75] showed that SGLT2i use was associated with a 30% reduction in incident gout events/gout flares (HR 0.70, 95% CI: 0.59, 0.84, *p* < 0.001).

### Electrolyte disorders

#### Hyponatremia

Hyponatremia related to secondary ADH secretion is present in ~ 5% of all patients suffering from HF on diuretics. The risk of hyponatremia is especially high for thiazide diuretic users, and within this subclass higher for chlorthalidone than for hydrochlorothiazide [36]. Thiazide-associated hyponatremia generally occurs in the first 1.5 weeks after thiazide initiation, but the risk remains elevated after this time-frame. Concomitant use of thiazide diuretics and SSRIs, venlafaxine, NSAIDs, loop diuretics and carbamazepine should be avoided, especially in older (≥ 75 years old) non-ambulatory individuals because of increased risk of hyponatremia [76]. Of note, thiazide-induced hypokalemia tends to aggravate hyponatremia as a result of shifting of sodium intracellularly. Hyponatremia contributes to fall risk through altered mental state, dizziness, gait disorders or muscle weakness, even if serum concentrations are only mildly decreased (< 135 mEq/L) [77, 78]. A recent study [79] demonstrated that the risk of falling in individuals having sodium levels < 125 mEq/L was five times higher compared to individuals with levels ≥ 125 mEq/L).

#### Hyperkalemia, hypokalemia and/or hypomagnesemia

Hyperkalemia is present in up to 40% of chronic HF patients [80] and results in arrhythmia-related fall risk. The potassium sparing diuretics predispose to hyperkalemia, especially in patients with chronic kidney disease and eGFR ≤ 30 mL/min per 1.73 m^2^. Concomitant use of spironolactone with ACEis/ARBs, amiloride, triamterene and potassium supplements increase the risk of developing hyperkalemia, especially in case of dehydration and chronic kidney disease. Literature suggests that SGLT2i use is not associated with incident hyperkalemia, not even in high-risk patients with type 2 diabetes mellitus and chronic kidney disease [81, 82].

Hypokalemia in chronic HF patients has been linked to increased mortality and risk of sudden death, syncope and falls [83], even when of mild severity (≤ 4.1 mEq/L) [17]. The risk of developing hypokalemia is highest for loop diuretics [65], but lower for torasemide compared to other loop diuretics [84]. Among thiazide(like) diuretics, the risk of hypokalemia is relatively high for chlorthalidone, moderate for hydrochlorothiazide, and low for indapamide SR (sustained release) [70]. Thiazide, but not loop diuretic use, is associated with hypomagnesaemia [85]. Chronic potassium and magnesium depletion may contribute to fall risk due to muscle weakness (e.g. loss of muscle strength, inability to stand up) and life-threatening arrhythmias [17, 86]. In addition, hypomagnesemia may negatively impact bone health, contributing to osteoporosis and osteomalacia [86].

### Weight loss and thiamine deficiency

An important effect of diuretics and SGLT2is to consider in geriatric patients with a history of falls is the potential to cause weight loss [48]. After initiating these medications, weight loss is observed (for SGLT2is, weight loss due to caloric loss is ~ 2–3 kg, plateauing in 3–6 months). Not only is a lower body mass index an independent fall risk factor for falls in older adults [87], it is also associated with OH [88]. In addition, low BMI is an independent risk factor for all-cause mortality and HF hospitalization in older (≥ 75 years old) HF patients [89].

Chronic use of high doses of loop diuretics (except torasemide [84]) are associated with thiamine deficiency [17]. Low thiamine levels are associated with lower left ventricle ejection fraction and wosening HF symptoms [84] and should, therefore, be monitored and treated if necessary in older adults with poor dietary intake.

### Sedation and anticholinergic potential

Patients with hepatic impairment, cirrhosis and ascites are at increased risk for dehydration-associated hepatic encephalopathy [90], which may increase fall risk. Therefore, diuretics should be prescribed with caution in fall-prone older adults with these comorbidities. According to the Anticholinergic Drug Scale [91], anticholinergic effects have been demonstrated by a serum assay study for chlorthalidone, furosemide and triamterene, whereas bumetanide does not exhibit anticholinergic activity. The clinical relevance hereof, however, is questionable.

### Impaired hearing and vertigo

High intravenous and oral doses of loop diuretics (especially furosemide) are associated with impaired hearing, tinnitus and vertigo [31] and, therefore, inappropriately high diuretic dosing could contribute to fall risk [58]. The risk of ototoxicity may be lower with continuous intravenous loop diuretic infusion compared to bolus therapy [92].

### Bone quality and fracture risk

Several studies have demonstrated an association between fragility fractures and the use of diuretics in individuals ≥ 65 years old. Fracture risk was highest in the first weeks after initiating diuretic therapy [40, 93, 94]. Hip fracture risk was highest in the first week after initiation of loop diuretics (OR = 1.1; 95% CI: 0.7, 1.9), and in the second week following thiazide diuretic initiation (OR = 2.2, 95% CI: 1.2, 3.9) [95]. Incidence rate ratio (IRR) for loop diuretics was 1.74 (CI 1.61–1.89), and 1.41 (CI 1.04 to 1.16) for thiazide diuretics (IRR, *p* < 0.01). Hip bone mineral density (BMD) was lower in chronic long-term loop diuretic users compared to nonusers (*p* = 0.03) [96]. Also in a large real-life older population, increased hip fracture risk was observed after initiating loop diuretic therapy (1.5 increased risk in patients aged 70–90 years; 1.8-fold increased risk in patients aged 81–90 years) [97].

The risk of bone fractures associated with SGLT2i use is controversial [48]. In the Canagliflozin Cardiovascular Assessment Study (CANVAS) trial, the incidence rate (IR) of bone fractures among those taking canagliflozin was significantly higher than that among those taking placebo (15.4 vs 11.9 fractures per 1000 person-years; hazard ratio [HR], 1.26; 95% CI, 1.04–1.52). This increased fracture risk, however, was not observed in other large RCTs of canagliflozin or other SGLT2is [38, 98, 99]. Post-marketing safety surveillance data are required to determine the effects or SGLT2is on bone health and fracture risk. Until then, a pragmatic approach would be to monitor parathyroid hormone (PTH) levels of patients on SGLT2is and to initiate vitamin D analogs if PTH levels are elevated [48].

## Deprescribing diuretics and SGLT2is

**Fig. 2:**
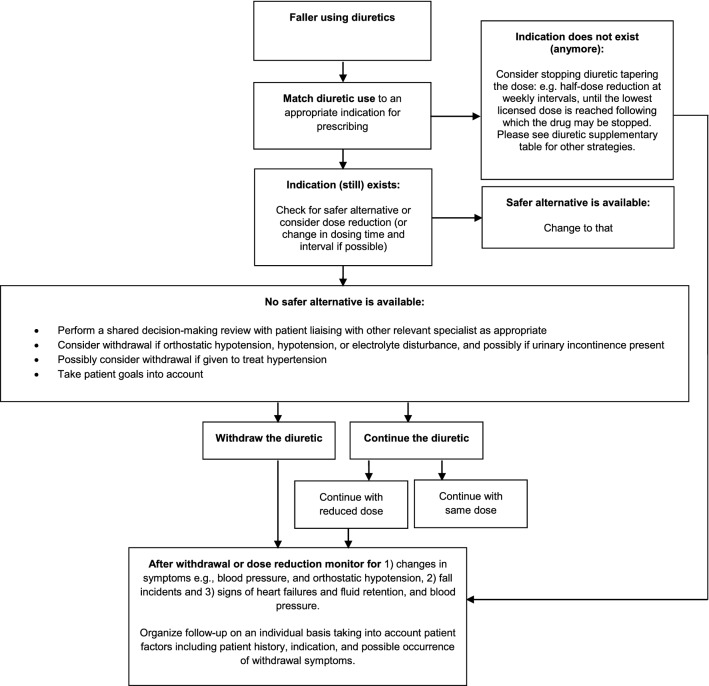
Clinical decision tree for diuretic withdrawal in patients who have fallen (for interactive version see https://kik.amc.nl/falls/decision-tree/)

In patients who have fallen, deprescribing diuretics and/or SGLT2is should always be considered and discussed with the patient, especially when fall-related adverse drug effects have been identified. The first step in diuretic users is to check whether dose-reduction can be attempted, for instance in individuals with poor dietary intake or signs/symptoms of dehydration. For diuretics, a step-by-step deprescribing approach for clinical use has been developed recently (Fig. 2, also for interactive use: https://kik.amc.nl/falls/decision-tree/). In the next subparagraphs, the evidence of deprescribing these drug classes is summarized, and recommendations for successful deprescribing outlined.

### Diuretic (and SGLT2i) deprescribing trials

Several diuretic withdrawal studies have been published, generally showing that the success rate of diuretic withdrawal is generally high (50–100% of participants). Recurrence of HF (present in approximately one in four patients) was the most common reason for failed withdrawal attempts [100]. It should be emphasized, however, that the value of this evidence for current clinical practice is highly questionable: the majority of trails was performed decades ago [101, 102] in relatively healthy, young, predominantly male HFrEF patients which is in sharp contrast to the contemporary real-world HF populations [103]. The studies were generally small, open-label observational studies of low to moderate quality and unclear or high risk of bias [100]. In some studies, patients with a history of HF were even excluded [11, 104]. Also, HF care was of low quality compared to contemporary care with evidence-based disease-modifying drugs and multidisciplinary HF outpatient programs [103]. Literature suggests that patients treated for HF according to the current guidelines are less prone to develop HF upon deprescribing diuretics [44, 60].

Failure of deprescribing attempts will generally be evident within the first weeks to months. After that time-frame, the risk of re-initiating diuretic therapy is low [10]. Factors predicting successful diuretic withdrawal are (a combination of) furosemide dose ≤ 40 mg/day, left ventricular ejection fraction > 0.27 and absence of a history of systemic hypertension [105]. If none of these criteria were present, the success rate was 71% after 6 weeks of intervention; if, however, all criteria were met, the probability of successful diuretic cessation was close to zero. Another recent hypothesis-forming paper based on post-hoc data from older patients with chronic HF [106] showed that repeated measurements of blood biomarkers (e.g., interleukin-6 (IL6), high-sensitivity C-reactive protein (hsCRP), blood urea nitrogen and prealbumin) may aid clinicians in safe diuretic dosing decisions. For example, high-dose loop diuretics were associated with low short-term (1 month) risk of HF hospitalization or if the inflammation markers IL6 and hsCRP were high, whereas the risk was high if inflammation levels were low. In the latter situation, dose lowering or discontinuation of diuretics may be appropriate.

Based on (scarce) published data, blood pressure may increase after diuretic withdrawal, but that this generally is of mild to moderate severity, and temporary in nature. In a recent Norwegian study [24], the effect of deprescribing antihypertensive therapy (including diuretics) on blood pressure values in nursing home patients was assessed. Although deprescribing antihypertensives initially increased systolic blood pressure from baseline 128 ± 19.5 mmHg to 143 ± 25.5 mmHg at four months after deprescribing, blood pressure returned to baseline values (mean 134 mmHg) at follow-up after 9 months.

To the best of our knowledge, there is no published data on deprescribing SGLT2is in older patients.

### Patient-centered approach

A recent study in older HFpEF patients [107] showed that the majority (over 90%) of patients would be willing to have one or more medicines deprescribed if their doctor deemed this possible, and that they would like to be involved in decisions about their medicines (92%). To maximize success rate of deprescribing attempts, a very thoughtful and individualized approach should be adopted, considering the patients’ care objectives [54]. Deprescribing decisions should be made after exploring the patients’ treatment goals, preferences, wishes and reluctance to deprescribing. In patients with cognitive impairment, the patients’ caregiver should be involved in deprescribing decisions and the monitoring phase. It should be realized that what matters most to patients is not static, but may shift over time. In fact, half of older falls patients preferred a decrease in fall incidence over a decrease in cardiovascular risk [108]. In goals of care discussions, the doctor should carefully explain that the benefit/harm balance of the drug proposed to deprescribe may have shifted over time for the patient. If the doctor fails to adequately explain, the patient may perceive the proposal to deprescribe medications that were used effectively in their younger years for long periods of time as inadequate, and may feel they are being “given up on” [7]. If the patient/physician relationship is at stake, deprescribing efforts will probably not be successful. Also, patients should be reassured that they will be monitored [109] for signs and symptoms of HF, and that diuretics and/or SGLT2is will be re-initiated (or up-titrated to the lowest effective dose) if necessary.

### Monitoring phase

Diuretic deprescribing attempts require careful monitoring for re-occurrence of congestion. Like in ambulatory HF care, patients should be encouraged to measure their body weight daily, and to alarm when their body weight increases and/or signs or symptoms of HF arise [17, 80]. Early diagnosis of impending decompensation in older patients is notoriously challenging [17] as commonly used clinical signs of congestion, NT-pro BNP and echocardiography may fail to identify decompensation [110]. Various cardiac telemonitoring devices are currently being tested for their feasibility in detecting worsening HF at an early stage, and trial results seem promising [80, 111]. Theoretically, these devices could be of value in preventing overt decompensation and HF related hospitalization in older HF patients in whom diuretic therapy is withdrawn.

Given the high baseline prevalence of (orthostatic) hypotension in older HF patients [88], and the fact that withdrawing antihypertensives has the potential to lower postural blood pressure drops [65] blood pressure in older fallers using these drug classes should be evaluated regularly. White coat effects should be evaluated, and the orthostatic reaction should be assessed according to the guidelines, for which we refer the reader to Table 1. This table sums up practical tools and resources that may assist clinicians treating older HF patients in rational, patient-centered (de)prescribing diuretics and SGLT2is (e.g. a detailed practical decision-tree for deprescribing diuretics in older adults who have fallen [54]; https://kik.amc.nl/falls/decision-tree/).

## Conclusions

Older HF patients are a highly complex and vulnerable population. If they experience unexplained, recurrent falls, referral to a dedicated falls and syncope expert center should be considered to assess likeliness of cardiac (pre)syncope, and identify potentially modifiable fall risk factors such as FRID use: older HF patients are not only at risk for cardiac syncope, but also at very high fall risk due to both high prevalence of fall risk increasing conditions and multimorbidities (e.g., frailty, depression, and dementia). The use of diuretics and/or SGLT2is, drug classes with various potential fall-related adverse effects add to the increased fall risk in this population. Because falling is an important risk factor for functional decline, loss of quality of life and mortality, falls prevention is of great importance to older HF patients. To be effective, an individualized, patient-centered multifactorial treatment should be adopted, in which all factors contributing to fall risk in a patient should be adequately addressed.

Given the potential to cause or aggravate fall risk, diuretics and/or SGLT2is should be prescribed with caution and in the lowest dose necessary to maintain euvolemia in older fall prone HF patients. During regular follow-up visits, volume status, blood pressure and orthostatic response should be assessed. Also, regular evaluation of electrolytes is indicated, particularly in frail patients with poor dietary intake and/or risk of dehydration.

In carefully selected fall-prone older HF patients, diuretic withdrawal may be attempted in an effort to reduce fall risk. Careful monitoring of impending congestive HF and hypertension, however, is mandatory. Switching to SGLT2i therapy may be an attractive treatment option in some patients [29]: SGLT2is demonstrate beneficial cardiovascular outcomes, and relatively safe side effect profiles, even in older patients. Owing to their pleiotropic effects, SGLT2is may even facilitate deprescribing of potentially harmful co-medications (e.g., antihypertensives and antidiabetics) [48]. Yet, it should be emphasized that SGLT2is are a relatively new drug class for which clinical experience is still limited and long-term safety data are scarce, especially in frail older adults. Based on a very recent review article evaluating the risk–benefit profile for the use of SGLT2 inhibitors in this population [29], however, and based on the current evidence base, the benefits of SGLT2i use appear to outweigh the associated harms. For now, judicious use and strict monitoring in this patient group remains mandatory.

Decisions to (de)prescribe diuretics and SGLT2is in older HF patients are highly complex and challenging to clinicians. This paper is aimed to support clinicians in decision making regarding continuation/deprescribing diuretics and SGLT2is: we summarized the best available evidence from the literature to safely prescribe and deprescribe these drug classes and provide the clinician with guidance and practical tools to be used in everyday clinical care for older HF patients who have fallen. It should be emphasized that falls are usually multifactorial in nature. To effectively reduce fall risk, a multifactorial and comprehensive approach should be adopted, in which all fall risk factors—including risk medication— present in a patient are addressed [1, 112]. For detailed guidance on the multifactorial fall preventive approach, we refer to the recently developed World Falls Guidelines [1].

## Back to the case illustration

An important goal of care for this 83-year-old lady is to prevent future fall incidents and to prevent hospital admission. To this end, all identified (modifiable) fall risk factors should be addressed and discussed with her. Deprescribing furosemide and hydrochlorothiazide should be considered because these drugs may have caused nocturia, (orthostatic) hypotension, dehydration, azotemia, and hyponatremia. For practical guidance, the clinician can follow the STOPPFall clinical decision tree on deprescribing diuretics (Fig. 2, and https://kik.amc.nl/falls/decision-tree/). For this patient, SGLT2i therapy may be a safer alternative to diuretic use; compared to diuretics, tendency to cause electrolyte abnormalities of SGLT2is is low, and effect on blood pressure and diuresis is relatively weak. Also, whereas diuretics are only effective in treating/preventing congestion, SGLT2is have disease-modifying effects, linked to reductions in heart failure hospitalization and MACE outcomes. Last, introducing SGLT2i therapy will allow insulin dose reduction or even insulin cessation and, therefore, hypoglycemia risk. A safe approach to switch the patient from diuretic to SGLT2i therapy would be to cross-titrate (gradual diuretic dose reduction and introducing SGLT2i therapy), while carefully monitoring for signs/symptoms of congestion and increases in blood pressure. If serum creatinine increases upon initiating SGLT2i therapy, the clinician can use the recently published practical recommendations for dealing with (pseudo)worsening of kidney function after starting SGLT2is in heart failure patients [47]. To further mitigate fall risk, the patient should be advised to limit or stop alcohol ingestion, and to avoid benzodiazepine use. She should receive patient information on orthostatic hypotension, including counter maneuvers she can apply upon taking a standing position after laying or sitting, and sick day rules. Last, a (multidisciplinary) fall preventive program could be offered to her, aimed at increasing her physical activities and reducing her fear of falling.

## Data Availability

Not applicable.

## Abbreviations

ACEi: Angiotensin-converting enzyme inhibitor
ARB: Angiotensin receptor blocker
ARNI: Angiotensin receptor-neprilysin inhibitor
CCB: Calcium channel blocker
CKD: Chronic kidney disease
FRID: Fall-risk-increasing drug
HF: Heart failure
HFpEF: Heart failure with preserved ejection fraction
HFrEF: Heart failure with reduced ejection fraction
MACE: Major adverse cardiovascular event
MRA: Mineralocorticoid receptor antagonist
OH: Orthostatic hypotension
RAASi: Renin-angiotensin-aldosterone system inhibitor
SGLT2i: Sodium-glucose transporter 2 inhibitor
